# Identification and Functional Mechanism of Novel Angiotensin I Converting Enzyme Inhibitory Dipeptides from* Xerocomus badius* Cultured in Shrimp Processing Waste Medium

**DOI:** 10.1155/2018/5089270

**Published:** 2018-05-08

**Authors:** Xiujun Gao, Xiqi Li, Peisheng Yan, Rui Sun, Guangfeng Kan, Ying Zhou

**Affiliations:** School of Marine Science and Technology, Harbin Institute of Technology, Weihai, West Culture Road 2, Weihai, Shandong 264209, China

## Abstract

ACE inhibitory dipeptides from* Xerocomus badius* fermented shrimp processing waste were isolated with ethanol, macroporous resin, chloroform, and Sephadex G-10 in sequence and identified by LC-MS/MS system coupled with electrospray ionization source. Molecular docking was performed for exploring the mechanism of their inhibitions. The results showed that the identified ACE inhibitory dipeptides were Cys-Cys and Cys-Arg with IC_50_ values of 4.37 ± 0.07 and 475.95 ± 0.11 *μ*M, respectively. The difference between ACE inhibitor potency of Cys-Cys and Cys-Arg could be explained by results of molecular docking. Cys-Cys formed crucial coordination between carboxyl oxygen and Zn(II), hydrogen bonds with residues Ala354(O), Ala356(HN), and Tyr523(OH), and a bump with the residue His387(NE2) at the active site of ACE. There was no coordination, except for 5 hydrogen bonds (at residues His353, Ala354, Glu384, Glu403, and Arg522) and a bump (Glu411) between Cys-Arg and active site of ACE. These findings highlighted that Cys-Cys could be considered as a novel potent ACE inhibitor, and coordination between its carboxyl oxygen and Zn(II) played significant role in defining its ACE inhibitor potency.

## 1. Introduction

ACE, a zinc metallopeptidase activated by chloride, plays a pivotal role in the enhancement of blood pressure by converting angiotensin I to angiotensin II, which is a potent vasoconstrictor. Therefore, ACE inhibitors are primary drugs to treat hypertension [1, 2], a common cardiovascular disease, which is one of the major risk factors of several other cardiovascular diseases, such as stroke, coronary heart disease, and myocardial infarction. In recent decades, the most successful strategy to treat hypertension is the inhibition of ACE. However, routinely consumed ACE inhibitors used as antihypertensive drugs in clinical prescriptions including captopril, lisinopril, and alacepril [3, 4], are all synthetic. These drugs exhibit various side effects such as allergic reactions, cough, skin rashes, and taste disturbances. As a result, there is a great demand for safer and more compatible natural ACE inhibitors for treating hypertension in a nontoxic manner.

Therefore, ACE inhibitors from natural sources have been increasingly concerned as potential antihypertensive drugs. Many ACE inhibitory peptides from animal, plant, and microbe, such as seaweed pipefish [5], grass carp [6], silkworm pupa* (Bombyx mori)* [7], tilapia [8], arachin [9], and* Saccharomyces cerevisiae *[10], have been isolated and identified in the last two decades. ACE inhibitory activities of these peptides, which were revealed to be di- to tridecapeptide, were found to be dependent on their amino acid composition, structure, and hydrophobicity [11, 12]. Besides, molecular docking simulation is more and more used in exploring the domains of ACE inhibitory peptides binding with ACE for explaining their functional mechanism and evaluating potential antihypertensive medicines [7, 13–15].

In recent years, bioactive peptides isolated from fruiting bodies of many kinds of medicinal mushrooms have shown significant ACE inhibitory activities [16–19]. However, there has been few reports about the ACE inhibitory peptides from liquid fermented products of medicinal fungi, although it has the advantages of lowering the cost, making industrialization and control of product quality easier. In our previous studies, shrimp processing waste was fermented by* Xerocomus badius* and Angiotensin I converting enzyme (ACE) inhibitory fragments were obtained from the resulting products in the process of recovery chitin and antioxidant polysaccharides [20–22].

Shrimp processing waste is the solid waste in the form of head and body carapace, which comprises 45~60% of the whole shrimp [23]. So, a large majority of shrimp processing waste, with calcium carbonate, protein, chitin, minerals, and carotenoids as the major fractions, is being produced annually all over the world. The resources of these valuable components have been increasingly concerned in the purpose of economical and environmental performance. However, shrimp processing waste was mainly recycled as fertilizer and feedstuff in the early years [24–26]. Recently, extraction and character of chitosan [27], astaxanthin [28], carotenoids [29], and chitin [27] were more and more studied. During this extraction, deprotein is an indispensable work [30], whereas chemical methods for recovering protein may cause secondary pollution and increasing of the cost because of using large quantities of strong acid or base [31]. Additionally, the recycled proteins have an unpleasant smell. Thus, fermentation with lactic acid bacteria has been used for recovery of proteins in the process of extracting chitosan, astaxanthin, carotenoids, and lipids [32, 33]. Moreover, the recycled proteins reached the standard of human food and had various bioactivities [34, 35]. Fermenting shrimp processing waste with* X. badius* for ACE inhibitors can not only lay the foundation for the utilization of shrimp processing waste with high added value but also provide a new way to gain nontoxic ACE inhibitors inexpensively.

However, ACE inhibitors from mycelium of* X. badius* cultured in shrimp waste medium have not been identified; their functional mechanisms have not been revealed, although these findings have many potentially favorable consequences for gaining potent antihypertensive drugs and exploring their structure-activity relationship. Therefore, the ACE inhibitory peptides from* X. badius* fermented shrimp processing waste were separated and identified in this study. Interaction between ACE and peptides were investigated through molecular docking simulation for clarifying their functional mechanism of their ACE inhibition.

## 2. Materials and Methods

### 2.1. Experimental Materials and Strains

Shrimp processing waste was collected from a local shrimp processing plant and pulverized (the particle size is less than 80 mesh) after being dried at 50°C. Medicinal fungus* X. badius* was preserved in our laboratory.

### 2.2. Fermentation of Shrimp Processing Waste with* X. badius*

Shrimp processing waste was fermented with* X. badius* under the optimized conditions determined in our previous report [20]. The strain was inoculated (5% amount of liquid seeds fermented in PDA) with 100 mL liquid medium (containing 12.4% of shrimp processing waste powder, 1.0% of bran powder, and 1.1% of glacial acetic acid) on a shaking incubator at 120 rpm and 25°C for 3 days.

### 2.3. ACE Inhibitory Activity Assay

The ACE inhibitory activity assay was carried out with the RP-HPLC method mentioned by Hyun and Shin [37] with minor modifications. Sample solutions were prepared in various concentrations by dissolving in 50 mM HEPES buffer containing 360 mM NaCl at pH 8.3. ACE from rabbit lung was purchased from Sigma, St. Louise, MO, USA, and dissolved in double distilled water in ice bath at concentration of 250 mU/mL. Hip-His-Leu (HHL) was purchased from Sigma, St. Louise, MO, USA, and dissolved in the HEPES buffer at concentration of 0.3%. In the assay, 30 *μ*L of sample solution was mixed with 30 *μ*L of ACE and preincubated at 37°C for 5 min, and then the mixture was incubated with 90 *μ*L of HHL at the same temperature for 50 min. Finally, 100 *μ*L of 1 M HCl was added in the mixture for terminating the reaction.

The resulting hippuric acid was determined by RP-HPLC on a Varian C18 column (4.6 × 150 mm, 5u, Eka Nobel, Sweden). After 20 *μ*L of reaction product being injected, the column was eluted with the mobile phase (30% of acetonitrile water solution, containing 0.2% acetic acid) at the 1.0 mL/min of flow rate. The detection wavelength was 228 nm. ACE inhibition rate was formulated by the following equation:

(1) $$\begin{array}{c} \text{Inhibition rate (\%)}=\frac{\left({HA}_{C}-{HA}_{S}\right)}{\left({HA}_{C}-{HA}_{B}\right)}\times 100\%, \end{array}$$

where HA_*C*_ is the area of hippuric acid peak of control, HA_*S*_ is the area of the sample, and HA_*B*_ is the area of the blank group. The IC_50_ value was defined as the concentration of a certain sample required to inhibit activity of ACE (area of the hippuric acid peak) by 50%.

### 2.4. Purification of ACE Inhibitory Peptides

After the fermentation, the mycelium of* X. badius* was collected by filtering through two layers of gauze, flushed with distilled water to colorless and clear, lyophilized to constant weight, and weighed. The dried mycelium was pulverized, added to distilled water (1 : 50, w/v), and gently shaken at 50°C for 200 min. And then the resulting sample was centrifuged at 10,000 rpm for 10 min and filtered with filter paper for collecting the filtrate. The resulting extracted filtrate was vacuum-concentrated and dialyzed using MWCO 100 dialysis bags at 4°C for 12 hours.

The dialysate was diluted with three times their volume of ethanol (95%). Then the filtrate was collected by centrifuging at 10,000 rpm for 20 min, vacuum-concentrated, and adsorbed with D3520 macroporous adsorption resin (nonpolar, the average pore size of 85~90 Å, particle size of 60~16, Tianjin Haoju Resin Technology Co. Ltd.). After the adsorption, the most active fraction was mixed with chloroform at the same volume, shaked for 1 h, and allowed to stand for 30 minutes to separate into two layers. Then the most active fraction was further purified using Sephadex G-10 column eluted with 10 mM Tris-HCl buffer (pH 8.0) at a flow rate of 12 mL/h. The fractions were collected with a fraction collector at 15 min intervals, and the absorbance of every fraction was tested at 220 nm.

ACE inhibitory activity assays were performed after every separation step for tracking the active component. The fraction showing the highest inhibitory activity ACE was further analyzed by LC-MS/MS.

### 2.5. LC-MS/MS and Peptides Synthesis

The amino acid sequence of the fraction was identified by using a LC-MS/MS system (Xevo-tqd, WATERS, USA) coupled with electrospray ionization (ESI) source. The sample was dissolved in methanol and separated by a UPLC using a BEH C18 column (4.6 × 150 mm, 3.5 *μ*m, 4.6 mm × 100 mm, WATERS, USA). To obtain spectra of intact and fragmented peptides, MS/MS spectra were acquired in data dependant MS/MS mode of collision cell.

Based on their results of LC-MS/MS, 2 dipeptides were chemically synthesized and purified (Ketai Biology Science and Technology Ltd., Shanghai, China). For control, Cys-Phe derived from shark meat hydrolysate, a potent ACE inhibitor with an IC_50_ value of 1.98 *μ*M [19], was synthesized and purified simultaneously. The synthesized peptides have the purity of more than 98% according to results of HPLC analysis. The molecular mass of the synthesized peptides was determined by using LC-MS/MS. The inhibitory activity on ACE of each synthesized peptide was determined with the method described above.

### 2.6. Stability of ACE Inhibitory Peptides

The purified dipeptides (4 mg/mL) were incubated at 20, 40, 60, 80, and 100°C separately for 2 h and then allowed to cool to room temperature (25°C) and the pH was adjusted to 8.3. ACE inhibitory activity of them was tested with the method described above. Solution of the two dipeptides stored at 4°C assayed their ACE inhibitory activity for control.

Assays of gastrointestinal digestion in vitro were assessed on the ACE inhibitory peptides for challenging their stability. Each peptide solution (4 mg/mL) was mixed with 0.1 M KCl-HCl (pH 2.0) buffer containing 1% (w/w) pepsin. The mixture was incubated at 37°C for 4 h and then 2 M NaOH solution was added to neutralize to pH 7.0. Then the 1 mL of each neutralized suspension was centrifuged at 8,000 rpm for 20 min and the supernatants were assayed for ACE inhibitory activity. Meanwhile, the remaining neutralized suspensions were further digested by pancreatin (1%, w/w) at 37°C for 4 h and boiled for 15 min to terminate the pancreatic digestion. Then, the reaction mixtures were centrifuged at 8,000 rpm for 20 min and the supernatants were used for ACE inhibitory activity assays. The ACE inhibitory peptides were mixed with inactivated pepsin and pancreatin boiled for 15 min and assayed for ACE inhibitory activity for controls.

### 2.7. Molecular Docking of the Peptides on the ACE Binding Site

The structure of each identified ACE inhibitory peptide was constructed using ArgusLab 4.01 and its energy was optimized using Discovery Studio (DS, Accelrys, San Diego, CA) 2.5. The crystal structures of human ACE-lisinopril complex (1O86.pdb) were derived from the RCSB PDB Protein Data Bank (http://www.rcsb.org/pdb/home/home.do). Before docking, the ACE model was prepared by removing water molecules and the inhibitor ligand, retaining the cofactors zinc and chloride atoms, and adding the polar hydrogen. Docking simulation was performed using Flexible Docking tool of DS 2.5 software.

### 2.8. Statistical Analysis

All the experiments were performed in triplicate, and data were analyzed by using the SPSS 16.0 software. The 95% confidence interval (*P* < 0.05) was determined as significant differences.

## 3. Results

### 3.1. Isolation and Purification of ACE Inhibitory Peptides

Inhibitory activities of all fractions on ACE were tested for tracing the potent ACE inhibitors. The active fractions and their IC_50_ values were shown in Table 1.

Alcohol precipitation method was used to separate the water extract of mycelium from* X. badius* fermented shrimp processing waste (WEMXS) into two fractions, WEMXS-I (the extracted supernatant) and WEMXS-II (crude polysaccharide). In these two fractions, WEMXS-I exhibited higher ACE inhibitory activity (IC_50_ = 162.41 *μ*g/mL). And then, WEMXS-I was absorbed with macroporous resin (D3520) and separated into MRD-1 (free components) and MRD-2 (components absorbed by the resin), and MRD-1 was determined to have higher inhibitory activity on ACE (IC_50_ = 132.77 *μ*g/mL). Thus, MRD-1 was further separated by extracting with chloroform. The inhibitory activity on ACE of resulting aqueous phase (named as C1, IC_50_ = 146.27 *μ*g/mL) was slightly lower than the chloroform phase (named as C2, IC_50_ = 114.94 *μ*g/mL), but the difference was not significant. Furthermore, C1 showed purple in ninhydrin reaction, and its characteristic absorption wavelength was 220 nm, which implied that components in C1 may be made up of peptides. Therefore, C1 was selected for further purification by applying to the Sephadex gel chromatography, whereas C2 showed colorless in ninhydrin reaction, and its characteristic absorption wavelength was 260 nm. These indicated that components in C2 were not peptides. So, ACE inhibitors from C2 will be purified and identified in ulterior study.

**Figure 1:**
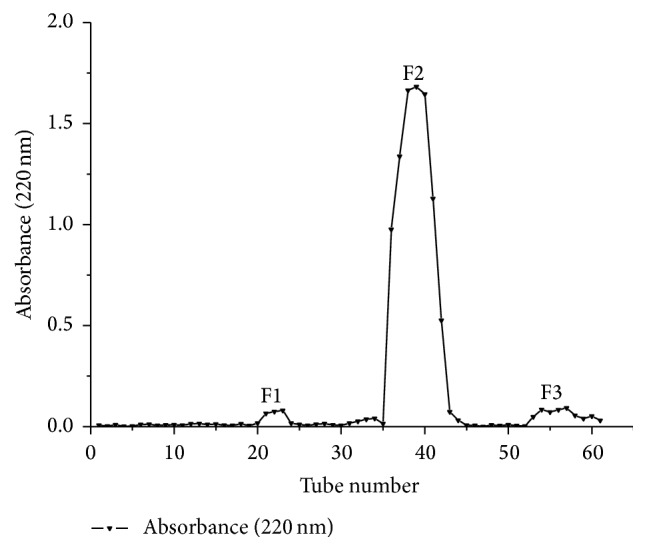
Purification profiles of ACE inhibitory peptide from C2 with Sephadex G-10 gel chromatography of fraction.

The active fraction C1 was further fractionated on a Sephadex G-10 gel filtration column and three peptide peaks were monitored at 220 nm (Figure 1). The second peptide peak was determined to have the strongest inhibitory activity on ACE (IC_50_ = 135.17 *μ*g/mL, named as F2). Amino acid sequence of F2 was identified through LC-MS/MS.

### 3.2. Amino Acid Sequence and IC_50_ Value of the Purified Dipeptide

**Figure 2:**
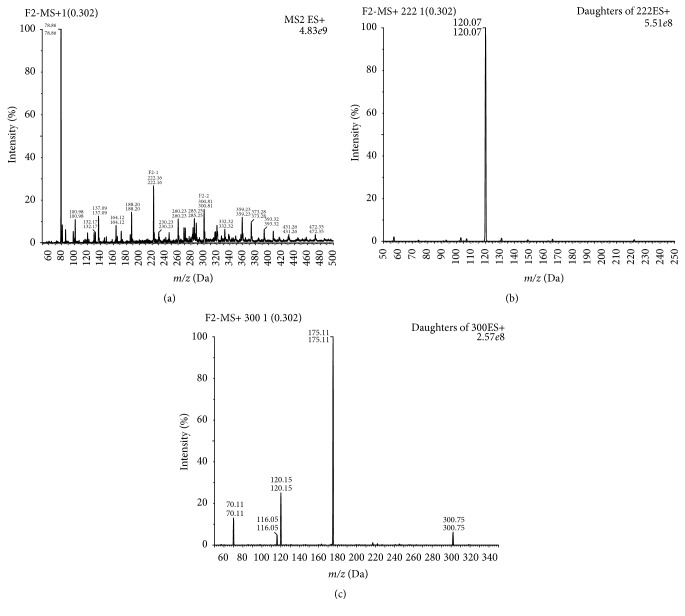
Molecular mass and amino acid sequence of potential ACE inhibitory peptide from the purified fraction F2. (a) IT-MS spectrum of purified F2 fraction. (b) IT-MS/MS spectrum of F2-1; (c) IT-MS/MS spectrum of F2-2.

The fraction F2 was separated and identified by LC-MS/MS. The mass spectra (Figure 2(a)) showed that molecular weights of peptides in F2 were all less than 500, and the ion peak of each peptide appeared at *m*/*z* of the theoretical value. Then, ion peaks of peptides with top 2 types of abundance (F2-1 and F2-2 shown in Figure 2(a)) were further identified by going through second ion mass spectrometry.

**Figure 3:**
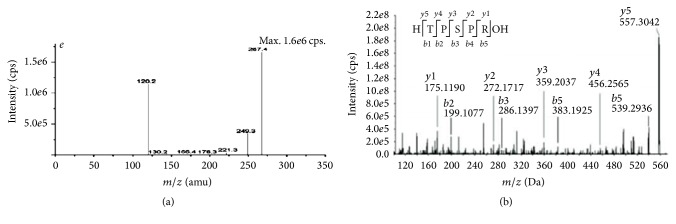
The second stage mass spectrum of Cys-Phe and Thr-Pro-Ser-Pro-Arg. (a) Cys-Phe [19]. (b) Thr-Pro-Ser-Pro-Arg [36].

As shown in Figures 2(a) and 2(b), *m*/*z* of amino acid composed F2-1 was 120.7 and was consistent with the report of Wu et al. shown in Figure 3(a) [19]. Accordingly, the amino acid was Cys, and F2-1 was Cys-Cys. The theoretical molecular weight of Cys is 121.2 and Cys-Cys is 224.3. Hence, the ion peak of F2-1 and the ion peak with 120.7 *m*/*z* were negatively charged.

As shown in Figures 2(a) and 2(c), *m*/*z* of amino acids composed F2-2 were 120.15 and 175.11, consistent with report of Wu et al. (Figure 3(a)) [19] and Yu et al. (Figure 3(b)) [36]. Accordingly, the amino acids were Cys and Arg, and F2-2 was Cys-Arg. The theoretical molecular weight of Arg is 174.8, and Cys-Arg is 277.3, so that the ion peak of F2-2 was positively charged and the ion peak with 300.81 of *m*/*z* was the sodium added peak.

Cys-Cys, Cys-Arg, and Cys-Phe were synthesized and their ACE inhibitory activities were determined. The results (Table 2) showed that Cys-Cys had the highest inhibitory activity on ACE (IC_50_ = 4.37 *μ*M), followed by Cys-Phe (IC_50_ = 13.82 *μ*M). Cys-Arg had the lowest ACE inhibitory activity with an IC_50_ of 475.95 *μ*M.

### 3.3. Stability of ACE Inhibitory Peptides

**Figure 4:**
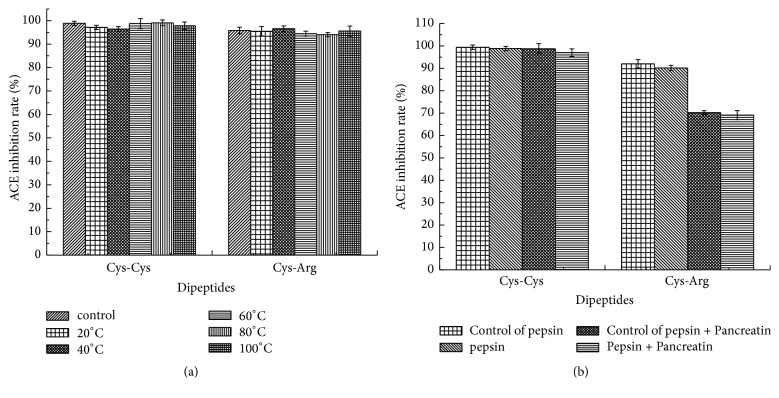
Thermo and gastrointestinal digestion stability of Cys-Cys and Cys-Arg. (a) Incubation at various temperatures. (b) In vitro gastrointestinal digestion.

The purified ACE inhibitory dipeptides, Cys-Cys and Cys-Arg, retained their ACE inhibition rate at every temperature selected (Figure 4(a)), which implied that dipeptides Cys-Cys and Cys-Arg were both thermostable. Additionally, the ACE inhibitory activity of dipeptides gastrointestinally digested in vitro was reduced slightly but not significantly compared with the control (Figure 4(b)). This suggested that dipeptides Cys-Cys and Cys-Arg were stable in gastrointestinal proteinases.

### 3.4. Insight into Molecular Docking Simulation

**Figure 5:**
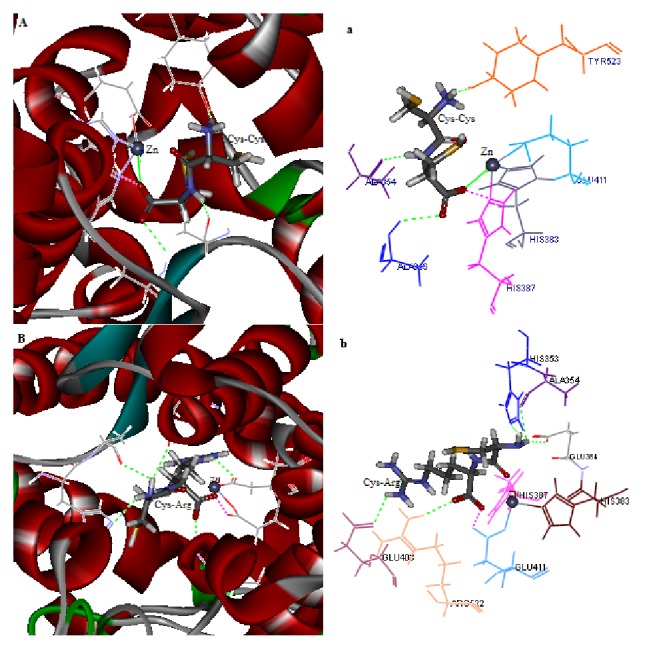
The best ranked docking pose of Cys-Cys and Cys-Arg with ACE (PDB: 1O8A). (A) Local overview of docking pose of Cys-Cys at the ACE active site. (a) The binding mode between ACE residues and Cys-Cys at the ACE active site (green dotted line indicates hydrogen bond formation, purple dotted line indicates bump formation, and green solid line indicates coordination). (B) Local overview of docking pose of Cys-Arg at the ACE active site. (b) The binding mode between ACE residues and Cys-Arg at the ACE active site (green dotted line indicates hydrogen bond formation and purple dotted line indicates bump formation).

The simulations of molecular docking of Cys-Cys and Cys-Arg binding with ACE were performed for exploring the structural-functional mechanism of the dipeptides inhibition on ACE by using Flexible Docking tool of Discovery Studio 2.5 software. The most stabilized docking poses have been shown in the ACE active site. After docking, Cys-Cys and Cys-Arg were buried deep inside the active site channel of ACE (Figures 5(a) and 5(b)) and combined with ACE residues through the interaction forces of hydrogen bonds, bump, and coordinate bonds. The binding sites and distances of these hydrogen bonds, bumps, and coordinate bonds were displayed in Table 3.

According to the results of the docking, the dipeptide Cys-Cys formed 3 hydrogen bonds with ACE residues Ala354, Ala356, and Tyr523, and a bump with His387. Furthermore, Cys-Cys is positioned to coordinate the active site Zn(II) atom, which binds to residues His383, His387, and Glu411. The distance between Zn(II) and the carbonyl oxygen of the Cys-Cys was calculated as 2.088 Å. These results indicated that the dipeptide Cys-Cys could effectively interact with the active site of ACE and all of the interactions might play important roles in ACE binding.

As to Cys-Arg, the results of the docking showed that 5 hydrogen bonds formatted between the dipeptides and ACE at Ala354, Glu384, His353, Glu403, and Arg522. Besides, Cys-Arg formatted a bump at Glu411. Some ACE inhibitors interact with Zn(II) directly and/or with residues His383, His387, and Glu411 [7, 9, 14, 15]. Although it had no coordination with Zn(II) directly, Cys-Arg formatted 5 hydrogen bonds with ACE at some residues belonging to subsite pocket S1 and S2, as well as 1 bump between dipeptide and Glu411. These interactions maybe cause distortion of the tetrahedrally coordinated Zn(II), therefore losing ACE activity.

## 4. Discussion

ACE inhibitory peptides from natural products, especially from medicinal fungi, are increasingly concerned with their prophylactic and therapeutic benefits on hypertension and having no harmful side effects. Many ACE inhibitory peptides derived from fruiting bodies of edible/medicinal fungi were identified [16–19]. But there are few reports about ACE inhibitory activity of liquid fermented products of these fungi, although this method makes the cost lower and industrialization easier. On the other hand, shrimp processing waste contains a large proportion of protein, which must be removed in the extraction of chitin and astaxanthin. However, recovering the waste protein for animal feed and fertilizer has low added value. In this study, high value added ACE inhibitory dipeptides from* X. badius* fermented shrimp processing waste were identified. These results provide not only the basis for full resource of shrimp processing waste with high added value, but also a new way to gain nontoxic ACE inhibitors inexpensively.

It has been shown that many natural short peptides contain 2–7 amino acids. It is also revealed that the majority of ACE inhibitory dipeptides have 0.92~130 *μ*M of IC_50_ values and are most likely to contain Phe or Tyr as the first residue from C-terminal [19]. These are different from the novel ACE inhibitory dipeptides, Cys-Cys and Cys-Arg, which are identified in this paper for the first time.

The differences in test conditions, ACE enzyme, and substrates used in the ACE inhibitory activity assay can result in the difference in IC_50_ values of the same sample. Cys-Phe had 1.98 *μ*M of IC_50_ value in previous reports, which were much lower than most of other ACE inhibitory dipeptides that have been reported [19]. ACE inhibitory IC_50_ value of purified Cys-Phe was determined as 13.82 *μ*M in the present paper, whereas IC_50_ value of purified Cys-Cys was determined as 4.37 *μ*M, which was much lower than that of Cys-Phe in this study. These results implied that the dipeptide Cys-Cys had much higher ACE inhibitory activity than most of other ACE inhibitory dipeptides that have been reported. The ACE inhibition IC_50_ value of purified Cys-Arg was determined as 34.44 times of Cys-Phe, which equaled moderate ACE inhibitory dipeptides.

Thermal stability of ACE inhibitory peptides was investigated in many related studies in order to gain accordance for technical process and storage requirement. In this study, the purified ACE inhibitory dipeptides, Cys-Cys and Cys-Arg, were both thermostable at selected temperatures for 2 h, which was similar to inhibitory peptides derived from tilapia [8]. Additionally, dipeptides Cys-Cys and Cys-Arg were stable in gastrointestinal proteinases. This finding was consistent with previous reports evaluating gastrointestinal enzymatic degradation of ACE inhibitory peptides [7, 38]. The present results showed that Cys-Cys and Cys-Arg were stable against pepsin, trypsin, and chymotrypsin, which could provide theoretical support for its practical application.

Application of molecular docking simulation made it possible to correlate the structure and activity of ACE inhibitory peptides and reveal the role of interaction forces between ACE and its inhibitor. ACE has three main subsite pockets named S1 including Ala354, Glu384, and Tyr523 residues, S2 including Gln281, His353, Lys511, His513, and Tyr520 residues, and S1' containing Glu162 residue [39]. It is reported that active site Zn(II) atom of ACE binds to residues His383, His387, and Glu411 to format a tetrahedral geometry and usually plays a significant role for ACE activity [40]. It is also reported that hydrogen bonds interaction force plays the most important role in stabilizing the docking complex and enzyme catalytic reactions [41]. Accordingly, the dipeptide Cys-Cys established hydrogen bonds with the subsite pockets S1 (Ala354 and Tyr523), which was similar to most of ACE inhibitory peptides reported [7, 9, 14, 15]. Besides, it is similar to Ile-Glu-Tyr and Ile-Glu-Trp that Cys-Cys established hydrogen bonds with Ala356 [9]. Furthermore, Cys-Cys is positioned to coordinate the catalytic Zn(II) directly, which could result in a decrease in IC_50_ value. Furthermore, the shorter the Zn(II) distance to the carbonyl oxygen of the peptide, the greater the degree of ACE inhibition [9]. The distance between Zn(II) and the carbonyl oxygen of the Cys-Cys was calculated as 2.088 Å, shorter than that of most ACE inhibitory peptides that have been reported [9]. This also explains why the dipeptide exhibits a better ACE inhibition than most of other ACE inhibitory peptides that have been reported. These results indicated that the dipeptide Cys-Cys could effectively interact with the active site of ACE and all of the interactions might play important roles in ACE binding.

It is conceivable that carboxylate docking, carboxy-terminal residue side chain interactions, and the coordination between carboxyl oxygen and Zn(II) and its distance are important in defining peptide ACE inhibitor potency [9, 41]. In this study, the most potent ACE inhibitor among the dipeptides is Cys-Cys (Table 2), which registered not only crucial coordination between carboxyl oxygen and Zn(II) but also interactions with ACE at Ala354 (O), Tyr523(OH), and His387(NE2). Substituting Cys in the carboxyl terminal with Arg restored a stronger hydrogen bond interaction between the inhibitor and Ala354(O) (2.314 Å), in addition to interactions at His353(NE2), Glu384(OE2)-H20, Arg522(HH11), and Glu403(OE1). Cys-Arg a 108.91-fold higher IC_50_ value was compared with Cys-Cys, although it registered more hydrogen bond and stronger bump with ACE at its active site. The important reasons are losing hydrogen bond at Tyr523(OH) (1.759 Å), crucial coordination between carboxyl oxygen and Zn(II), and bump at His387(NE2) (1.916 Å). Therefore, in these interactions, coordination between carboxyl oxygen and Zn(II) with distance of 2.088 Å played a significant role in high inhibitory activity of Cys-Cys on ACE.

Similar to previous reports, findings in this study highlighted the importance of coordination between carboxyl oxygen and Zn(II) and the interactions, especially the hydrogen bonds, between the inhibitory peptide and residues in active site of ACE in defining peptide ACE inhibitor potency.

## 5. Conclusions

Cys-Cys and Cys-Arg from* X. badius* fermented shrimp processing waste were successfully identified as novel ACE inhibitors. Cys-Cys has strong inhibitory activity on ACE, and Cys-Arg has moderate activity. The ACE inhibition of Cys-Cys is mainly attributed to forming crucial coordination between carboxyl oxygen and Zn(II). Cys-Arg formed 5 hydrogen bonds and a bump with residues in active site of ACE. Findings in this study highlighted the importance of coordination between carboxyl oxygen and Zn(II) in defining peptide ACE inhibitor potency. In vivo antihypertensive activity of the dipeptides will be evaluated in our further studies.

## Figures and Tables

**Table 1:** Purification of ACE inhibitory peptide of mycelium from *X. badius* fermented shrimp processing waste.

Purification step	Fraction	IC_50_ value (*μ*g/mL)	Purification fold
—	WEMXS	213.52 ± 15.44	1
Alcohol precipitation	WEMXS-I	162.41 ± 11.81	1.31
Macroporous resin (D3520)	MRD-1	132.77 ± 14.40	1.61
Chloroform extraction	C1	146.27 ± 16.28	1.46
	C2	114.94 ± 0.49	1.86
Sephadex G-10 gel	F2	135.17 ± 0.96	1.58

**Table 2:** Purified dipeptides and their ACE inhibitory activity.

Dipeptide	Molecular weight	Purity	IC_50_ value (*μ*M)
Cys-Cys	224.30	98.878	4.37 ± 0.07^*∗∗∗*^
Cys-Arg	277.35	98.8963	475.95 ± 0.11^*∗∗∗*^
Cys-Phe	268.34	99.0715	13.82 ± 0.09^*∗∗∗*^

*Note*. *∗∗∗* indicates the difference at *P* < 0.001 level from other dipeptides.

**Table 3:** Interaction between dipeptides and residues of ACE.

Cys-Cys		Cys-Arg	
Residue name	Distance (Å)	Residue name	Distance (Å)
Ala354(O)-H21^A^	2.337	His353(NE2)-H19^A^	2.367
Ala356(HN)-O12^A^	2.364	Ala354(O)-H20^A^	2.314
Tyr523(OH)-H16^A^	1.759	Glu384(OE2)-H20^A^	2.441
His387(NE2)-O13^B^	1.916	Glu403(OE1)-H34^A^	2.219
Zn-O13^C^	2.088	Arg522(HH11)-O18^A^	2.430
		Glu411(OE2)-O17^B^	1.548

*Note*. A refers to Hbonds, B to bump, and C to bonds.
